# Trends in *Polymers* 2017/2018: Polymer Synthesis

**DOI:** 10.3390/polym12010039

**Published:** 2019-12-25

**Authors:** Bernhard V.K.J. Schmidt

**Affiliations:** School of Chemistry, University of Glasgow, Glasgow G128QQ, UK; Bernhard.schmidt@glasgow.ac.uk; Tel.: +44-141-330-8469

**Keywords:** polymer synthesis, ionic polymerization, ring-opening polymerization, reversible deactivation-radical polymerization, coordination polymerization, emulsion polymerization, polymer composites, hybrid materials

**Polymer synthesis** is a substantial area in polymer science and marks the starting point for all sorts of polymer materials that have a plethora of applications in everyday life but also in academic research. Hence, polymer synthesis was chosen to be one of the seven sections in *Polymers* recently. In the years 2017 and 2018, a substantial amount of research articles and reviews have been published in *Polymers* addressing a broad range of synthetic methods, for example the traditional polymerization methods such as polycondensation [1], free radical polymerization [2], and photopolymerization [3]. Some of the major topics in polymer synthesis and significant contributions in *Polymers* are highlighted in the following article, i.e., the areas of ionic polymerization, reversible-deactivation radical polymerization (RDRP), ring-opening polymerization (ROP), coordination polymerization and polymerization in dispersed media as well as polymer composite and hybrid materials.

**Ionic Polymerization** methodologies embody a major way to produce well-defined polymer materials, for example, anionic ROP [4], carbanionic polymerization [5] or cationic polymerization [6]. In particular, living anionic polymerization can be utilized in combination with specific conjugation chemistry to produce polymer architectures as reviewed by Hirao and coworkers [7], e.g., block copolymers, star polymers and even more complicated architectures. Hadjichristidis and coworkers described the anionic polymerization of styrene and 1,3-butadiene employing phosphazene superbases [8]. Therefore, *sec*-BuLi was combined with various phosphazene bases forming highly reactive anionic species due to complexation with Li^+^. The best results were obtained with *t*-BuP_1_ with significant control over molar mass and molecular dispersities (*Đ*). More importantly, after changing the chain end to alkoxide via the introduction of 2–3 units of ethylene oxide (EO), ROP of cyclic esters could be performed in a one-pot reaction. Thus, ε-caprolactone (CL) or lactide (LA) were added to form block copolymers directly. Another combination of polymerization methods was described by Pitsikalis and coworkers [9]. At first, a norbornene containing alcohol was utilized as an initiator for anionic ROP of EO. In the next step, the formed PEO macromonomers were utilized in ring-opening metathesis polymerization (ROMP) to form a bottle brush polymer topology. The ROMP parameters like catalyst structure, length of macromonomer, catalyst and temperature were optimized to obtain well-defined brush polymers. Finally, the thermal properties of the polymer materials were studied. It was shown that the brush topology reduced the crystallinity of the PEO grafts and the thermal stability was enhanced due to the poly(norbornene) backbone. Recently, photopolymerization has gained significant attention [10,11], which has inspired Sangermano and coworkers to review the latest developments in cationic photopolymerization [12]. The authors focus on the synthesis of cationic photoinitiators, radical-induced cationic photopolymerization, and the frontal polymerization method.

**Reversible Deactivation-Radical Polymerization** (RDRP) is a highly relevant and convenient method for the synthesis of polymers with control over molar mass, end groups and *Đ* [13]. The common methods encompass atom transfer radical polymerization (ATRP) [14], reversible addition fragmentation chain transfer (RAFT) polymerization [15] and nitroxide-mediated polymerization (NMP) [16], which are used in polymer synthesis for various applications like drug-delivery, surface functionalization or organic electronics. In recent years metal-free RDRP approaches have been investigated frequently, which has been reviewed by Kreutzer and Yagci [17]. The review covers the common RDRP methods with special emphasis on mild polymerization conditions, e.g., via photo initiation. Li and coworkers described the visible light induced surface initiated ATRP from mesoporous silica particles (SBA-15) [18]. Therefore, methyl methacrylate (MMA), *N*-isopropylacrylamide and *N*,*N*-dimethylaminoethyl methacrylate were grafted from SBA-15 to fabricate inorganic-polymer hybrids employing 10-phenylphenothiazine as photo catalyst, where graft chain length could be tuned via polymerization time. The final materials could be employed for adsorption of organic molecules, e.g., toluene. Chen and coworkers reviewed advances in RAFT polymerization in particular [19]. In addition to photo-initiated RAFT polymerization, metal, enzyme, redox as well as acid governed initiation are summarized. Moreover, the recent development of sulfur-free RAFT polymerization is described. In terms of the application of RAFT polymerization, the authors emphasized the utilization of RAFT polymers in the field of optoelectronics. The RAFT copolymerization of styrene and *N*-phenyl maleimide (PMI) in 1,1,1,3,3,3-hexafluoro-2-propanol (HFIP) was investigated by Zhu and coworkers [20]. In comparison to the toluene (reactivity ratio PMI 0.078, styrene 0.068), considerably different copolymerization parameters were observed in HFIP (reactivity ratio PMI 0.026, styrene 0.050), i.e., a less alternating tendency of the copolymerization was observed. This behavior was ascribed to the hydrogen bonding between PMI and HFIP leading to additional steric repulsion as shown by computer simulations also. The formation of hyperbranched polymers via RDRP was reviewed by Gao and coworkers [21]. More specifically, the review focusses on self-condensing vinyl polymerizations via the utilization of inimers (monomers containing vinyl and initiating group) or transmers (monomers containing vinyl and chain transfer group). Both homopolymerization and copolymerization were described as well as the polymerization methods ATRP, RAFT polymerization, and NMP.

**Ring-opening Polymerization** (ROP) constitutes a considerable part in current polymer synthesis and is applied for the synthesis of various polymer types, e.g., polyesters, polyamides or polyethers. Especially the synthesis of PLA and PCL have been in the focus of research [22,23,24], which can be explained with their favorable mechanical properties, recyclability, and degradability [25,26,27]. Naturally, it has been frequently discussed in *Polymers* as well. Liu et al. synthesized aluminium complexes based on 8-anilide-5,6,7-trihydroquinone ligands for the ROP of cyclic esters [28]. The ROPs of CL and LA were investigated showing good control over molar mass and narrow *Đ*. Polyester nano bioglass composites were synthesized by Bikiaris and coworkers [29]. Therefore, CL was polymerized via ROP in the presence of nano bioglass that was fabricated via the hydrothermal method. Compared to neat PCL higher molar masses were obtained as well as improved mechanical strength of the polymer material. Moreover, the degradation was accelerated and bioactivity was enhanced due to the presence of the bioglass. A combination of RAFT polymerization and ROP was described by Huang and coworkers [30]. The authors described a copolymer of 3-ethyl-1-vinyl-2-pyrrolidone and *N*-vinylpyrrolidone with hydroxy endgroup via RAFT polymerization as first block. The hydroxy group was utilized to grow PCL as the second block via ROP forming an amphiphilic block copolymer. Finally, thermosensitive micelles were obtained. In addition to cyclic esters, i.e., lactones, the compound class of cyclic amides, i.e., lactams, are frequently polymerized via ROP as well, which gives rise to another important polymer class—polyamides. Thus, Karger-Kocsis and coworkers reviewed the ROP of lactams [31]. The review covers polymerization methodology, polymer types, and composite materials as well as manufacturing techniques. Another polymer calls, namely polyphosphates, were studied by Nifant’ev, who investigated the formation mechanism of polyphosphates in-depth [32]. More specifically, poly(ethylene phosphate) formation from cyclic ethylene phosphate monomers employing 2,6-di-*tert*-butyl-4-methylphenolate magnesium alkoxy complexes was investigated. Density functional theory showed that the initial dimeric magnesium complexes preferably act via a mononuclear pathway in the polymerization due to association with the monomer. Yagci and Kiskan described the ROP of 1,3-benzoxazines via borane catalysts [33]. Notably, the catalyst decreased the on-set polymerization temperature by 98 °C. Moreover, the thermal properties of the final polymer material were enhanced, e.g., the char yield was improved by 13%. The catalyst showed considerable solubility in the polymerization mixture and might be a metal-free alternative for benzoxazine resin formation for future applications. Paraskevopoulou and coworkers investigated the ROMP of norbornene and norbornadiene [34]. Therefore, a cis-selective bimetallic W-catalyst was utilized. The polymer structure could be tuned via a sequenced monomer addition and a comonomer ratio.

**Coordination Polymerization** belongs to the most important technical processes in polymer synthesis, mostly in the synthesis of polyolefins. As such, new developments are closely related to applications in polymer technology. A major focus in coordination polymerization is the development of new transition metal catalysts [35], e.g., giving rise to improved tacticity, activity or control over polymer architecture. Long and coworkers studied a sterically-demanding Ni(II) diimine catalyst for ethylene polymerization [36]. A living polymerization up to temperatures of 75 °C was achieved. Moreover, the polymerization could be turned “on” or “off” via monomer removal or addition. This effect was utilized to synthesize ethylene-based block copolymers, where the individual blocks were polymerized at different temperatures to change their branching density in the respective block. Bis(β-ketoamino)Ti(IV) complexes were utilized as catalysts for ethylene-norbornene copolymerization by Gao and coworkers [37]. Notably, the copolymerization activity obtained molar mass and norbornene incorporation relied strongly on the steric bulk of the ligands in the Ti complex. As such, the glass transition temperature could be tuned as well as the comonomer sequence. Pure poly(norbornene) was studied by Yao and coworkers [38]. Therefore, Ni(II) complexes with Schiff base ligands were employed that showed high catalytic activity when using methylaluminoxane as co-catalyst. The effect of ligand structure on the behavior in coordination polymerization of isoprene was studied in depth by Wang and coworkers [39]. More specifically, poly(isoprene) was synthesized with Fe(II) and Co(II)-derived catalyst complexes based on iminopyridine ligands. It was found that fluorinated ligands led to enhanced catalyst activity. Luo and coworkers studied the coordination polymerization of 1,3-butadiene catalyzed by rare-earth metal complexes via density functional theory [40]. A different kind of monomer was studied by Chen and coworkers [41], who had a look at poly(3-hexylthiophene) (P3HT) macromonomers with methacrylate end group. Chiral *ansa*-zirconocene catalysts were employed to synthesize P3HT brush polymers and copolymers with MMA. The polymerization yielded polymers with high stereoregularity. Furthermore, the syndiotactic helical polymer could be utilized to encapsulate C_60_ forming a supramolecular donor/acceptor complex that is considerable interest for organic electronics.

**Polymerization in dispersed media** has been in the focus of polymer synthesis for a long time and is still a major area of research [42,43,44], which is due to safety aspects, i.e., improved heat transfer, but also due to the opportunity of synthesizing well-defined polymer particles for various applications including photonics, bioimaging and drug-delivery [45,46,47]. Common methods are suspension polymerization or emulsion polymerization, as employed by Li and coworkers [48] as well as Guo and coworkers [49], respectively. Especially, the kinetics of emulsion polymerization of interest to ensure good control over the reaction and product properties. For example, vinylidene fluoride emulsion polymerizations were investigated by Beuermann and coworkers in a Monte Carlo approach [50]. Surfactant free emulsion polymerization has been of interest in the past years as it circumvents surfactant impurities as well as tedious work-up procedures and allows more efficient recycling of polymerization media [51,52], which was reflected in the work published in *Polymers* as well. A surfactant-free RAFT emulsion polymerization was described by Abetz and coworkers [53]. Well-defined PS latexes were obtained utilizing the RAFT-derived block copolymer poly(*N*,*N*-dimethylacrylamide)-*b*-poly(*N*-acryloylpiperidine-*co*-*N*-acryloylpyrrolidine) as thermoresponsive stabilizer. The block copolymers were tuned in a way that allowed the formation of micelles at polymerization conditions and polymerization of styrene inside of the micelle core at the same time. As such, latexes with thermosensitive corona were synthesized consisting of high molar mass PS block copolymers. Prasassarakich and coworkers used emulsifier-free RAFT emulsion polymerization to form poly(isoprene)-silica nanoparticles [54]. Therefore, silica nanoparticles were grafted with poly(styrene sulfonate sodium salt) via RAFT polymerization and finally with poly(isoprene) to obtain core-shell particles. The particle sizes were narrowly distributed and had a size in the range of 25 to 75 nm. Finally, the core-shell particles were blended with natural rubber, which led to improved reinforcement compared to plain silica particles due to improved compatibility of filler and matrix. Silica particles were utilized as a stabilizer by Zhang and coworkers in order to prepare polymer microspheres [55]. During the polymerization, malachite green was added as a template for molecular imprinting. For the work-up, the template was removed as well as the silica particles, which enables improved access to the molecular imprints. The polymer microspheres were subsequently utilized to adsorb malachite green from aqueous solution in a static environment and inflow via a column setup.

**Polymer Composites and Hybrid Materials** allow combining the features of non-polymeric materials, e.g., inorganic particles or biomaterials, with polymers [56,57,58]. On one hand, features like conductivity, catalytic activity or magnetic properties can be introduced to polymers via non-polymeric materials. On the other hand, polymers offer processability and improved mechanical properties to non-polymer materials. As such a mutual benefit is generated for both material classes. A major area is the combination of polymers with conductive materials [59]. Feng and coworkers performed electrochemical polymerizations of hydroquinone on graphite [60]. As such, poly(hydroquinone)-graphite composite films were obtained that showed pseudo-capacitative behavior. The films were utilized as anode material in microbial fuel cells, leading to increased power density. Also for the application of microbial fuel cells, Wu et al. combined poly(aniline) (PANI) with carbon nanotubes (CNTs) [61]. Therefore, an indium-doped tin oxide electrode was grafted with PANI. Next, a layer of CNT was added and the procedure repeated 2-3 times to obtain a PANI/CNT multi-layer structure on the electrode. After assembly of a microbial fuel cell, increased power density was observed compared to the bare electrode, albeit the negative effect of CNTs on cell viability has to be tackled in the future for improved long-term stability. A composite of the biomaterial cotton and PANI was described by Cavaco-Paulo and coworkers [62]. There, aniline was polymerized in the presence of cotton via an enzymatic pathway. Laccase together with 1-hydroxybenzotriazole as a mediator was utilized for the polymerization under mild reaction conditions to ensure the preservation of the cotton scaffold. Finally, conductive cotton fabrics were obtained that might be used for wearable electronics in the future. The composite formation has a significant influence on the thermal stability of polymer materials as well [63,64]. Achilias and coworkers studied the effect of Montmorillonite clay particles in the PMMA matrix on thermal properties and viscoelastic behaviour [65]. The composites were formed via free radical polymerization of MMA in a clay/MMA dispersion. The thermal degradation behaviour was studied via thermogravimetric analysis and showed a two-step behaviour. Moreover, increased stiffness was observed up to a Montmorillonite loading of 5 wt.%. In a similar way, the effect of graphene oxide on PMMA properties was studied, which showed improved thermal stability again [66]. Kadokawa and coworkers reviewed the enzymatic polymerization of saccharides around synthetic polymers [67]. As such, biopolymer/synthetic polymer composites can be synthesized with high precision.

**In summary**, polymer synthesis has marked its central position in polymer science and has been represented frequently in the Journal *Polymers* in 2017–2018. A plethora of polymerization methods was addressed as well as applications of the resulting polymer materials. We are looking forward to future developments and hope to find many of them freely accessible in *Polymers.*
